# Angiotensin-(1-7) ameliorates high glucose-induced vascular endothelial injury through suppressing chloride channel 3

**DOI:** 10.1080/21655979.2021.1997695

**Published:** 2022-02-15

**Authors:** Fei Cheng, Jing Liu, Zhuolin Guo, Shicheng Li, Jingfu Chen, Chang Tu, Fengzhou Fu, Bai Shen, Xiaojie Zhang, Guohua Lai, Jun Lan

**Affiliations:** a Second Ward of Cardiovascular Medicine, Dongguan Songshan Lake Center Hospital, Affiliated Dongguan Shilong People’s Hospital of Southern Medical University, Dongguan City, Guangdong Province, China; b Dongguan Cardiovascular Institute, Dongguan Third People’s Hospital, Dongguan City, Guangdong Province, China; c Second Ward of General Pediatrics, Dongguan Eighth People’s Hospital, Dongguan Children’s Hospital, Dongguan City, Guangdong Province, China

**Keywords:** Diabetes mellitus, cardiovascular disease, vascular endothelial injury, Angiotensin-(1-7), chloride channel 3

## Abstract

Diabetes Mellitus (DM) is a significant risk factor for cardiovascular disease (CVD), which is leading cause of deaths in DM patients. However, there are limited effective medical therapies for diabetic CVD. Vascular endothelial injury caused by DM is a critical risk factor for diabetic CVD. Previous study has indicated that Angiotensin-(1-7) (Ang-(1-7)) may prevent diabetic CVD, whereas it is not clear that Ang-(1-7) whether attenuates diabetic CVD through suppressing vascular endothelial injury. In this study, we found that Ang-(1-7) alleviated high glucose (HG)-induced endothelial injury in bEnd3 cells. Moreover, Ang-(1-7) ameliorated HG-induced endothelial injury through downregulating chloride channel 3 (CIC-3) via Mas receptor. Furthermore, HG-induced CIC-3 enhanced reactive oxygen species (ROS) and cytokine production and reduced the level of nitric oxide (NO), while Ang-(1-7) preserved the impact of HG-induced CIC-3 on productions of ROS, cytokine and NO through inhibiting CIC-3 via Mas receptor. Summarily, the present study revealed that Ang-(1-7) alleviated HG-induced vascular endothelial injury through the inhibition of CIC-3, suggested that Ang-(1-7) may preserve diabetic CVD through suppressing HG-induced vascular endothelial injury.

## Introduction

DM is a group of metabolic diseases characterized by hyperglycemia causing by impaired glucose tolerance, which is a significant risk factor for CVD [1–4]. Diabetic CVD is the leading cause of deaths in DM patients [5,6]. Although outstanding scientific advances have been made in understanding the factors contributed to CVD in patients with DM, there are limited effective medical therapies for CVD. Numerous studies have revealed that vascular endothelial injury caused by DM is a critical risk factor for diabetic CVD [7–10]. Damage of vascular endothelial cell by HG-induced inflammation, oxidative stress, cell apoptosis is the major cause of vascular endothelial injury [11–13]. Suppression of DM-induced vascular endothelial injury may provide a new therapy for diabetic CVD.

Angiogenesis is essential for wound healing not only in ischemic CVD but also diabetic CVD [14,15].The angiopoietin signal transduction system is the second most vital pathway for angiogenesis. Of the angiopoietins, angiopoietin 2 (Ang2) is an important marker of vascular endothelial injury associated to DM [14]. Previous studies have indicated that Ang2 enhances vascular permeability, destabilization and abnormal endothelial cell proliferation through counteracting the anti-inflammatory and pro-survival effect of Ang1 leading to vascular endothelial injury [16,17]. By contrast, Angiotensin- [1–7] (Ang- [1–7]) disintegrated from Ang2 protects against vascular endothelial injury through the effects of anti-inflammatory, antioxidation and anti-apoptosis [18–21]. Moreover, Ang- [1–7] may prevent diabetic CVD [22]. However, it is not clear whether Ang- [1–7] alleviates diabetic CVD through suppressing vascular endothelial injury.

As a member of the ClC voltage-gated chloride (Cl^−^) channel superfamily, chloride channel 3 (CIC-3) plays important roles in CVD through regulating cell proliferation, inflammation, volume regulation and apoptosis of vascular smooth muscle cells (VSMCs) [23]. For example, hypertension-associated vascular disease induces aberrant VSMC proliferation via upregulating CIC-3 expression [24]. Moreover, activation of CIC-3 channel in VSMC is required for myocardial hypertrophy and heart failure through promoting cytokine-induced reactive oxygen species (ROS) generation [25,26]. These studies suggest that CIC-3 may serve as an inducer of CVD. However, the functions of CIC-3 in diabetic CVD are unknown. Similar to the role in VSMCs, CIC-3 also regulates cell proliferation, apoptosis, inflammation and volume regulation of vascular endothelial cells [27–29]. Thus, CIC-3 may contribute to vascular endothelial injury in diabetic CVD.

In this study, we aimed to identify whether Ang- [1–7] attenuated diabetic CVD through suppressing vascular endothelial injury by CIC-3.

## Materials and method

### Cell culture and treatment

The bEnd3 cells were supplied by HeChuang Biotech, Inc (Guangzhou, Guangdong, China) and cultured by DMEM medium supplemented with 10% fetal bovine serum (FBS) in a humidified atmosphere of 5% CO2 with 95% air at 37°C. To set up a model of the HG-induced injury, cells were cultured in DMEM medium with 40 mM glucose (HG) for 24 h according to our previous study [30]. Besides, the cells of normal glucose group (NG) were cultured with DMEM medium supplemented with 5.5mM glucose. To investigate the protection of exogenous Ang- [1–7] against HG-induced injuries, cells exposed to HG were treated with or without Ang- [1–7] for 24 h. Transfection of Mas siRNA, CIC-3 siRNA and negative control siRNA (Hechuang Biotech, Inc) were performed using Lipofectamine 2000 (Invitrogen, Carlsbad, CA, USA).

### CCK-8 assay

1 × 10^4^ bEnd3 cells were seeded in a well of the 96-well plate and then the CCK-8 assay was employed to assess the cell proliferation. After the indicated treatments, 10 µl CCK-8 solution purchased from Beyotime Biotechnology (Shanghai, China) at a 1/10 dilution was added to incubate cells for 1.5 h at 37°C. Absorbance at 450 nm was assayed via a microplate reader (Molecular Devices, Sunnyvale, CA, USA). Subsequently, the optical density (OD) of cells were used to calculate the percentage of cell proliferation [31]. This experiment was carried out three times

### Immunofluorescence

First, bEnd3 cells were plated in slides covered on a 24-well plate and then fixed by 4% paraformaldehyde (PFA) for 1 h at room temperature (RT). After fixation, cells were treated with 0.2% Triton X-100 for 5 min at RT and washed by phosphate buffered solution (PBS) three times. Next, cells were blocked with 10% goat serum for 1 h at RT and the following incubation of anti-CIC-3 antibody (1:200 dilution) (#45,075 Signalway Antibody, College Park, MD, USA) in freshly prepared PBST (0.1 Tween20) with 1% goat serum at 4°C overnight. Cells were washed by PBST three times and incubated with fluorenes-conjugated secondary antibody (1:2000 dilution) in dark for 1 h at RT. After washing with PBST three times, slides were treated with mounting medium contained DAPI and took pictures by the BX50-FLA imaging system (Olympus, Tokyo, Japan). Next, the MFI (Mean fluorescence intensity) was measured by Image J 1.47i software as an index to the amount of CIC-3 expression. This experiment was carried out three times.

### Western blot (WB)

First, bEnd3 cells were harvested using a cell scraper and lysed by cell lysis solution at 4°C for 15 min. Next, the total proteins were quantified by the BCA protein assay kit (Beyotime Biotechnology). The same amount of protein from each sample was mixed with loading buffer and then boiled for 5 min followed by the fractionation using 10% sodium dodecyl sulfate-polyacrylamide gel electrophoresis (SDS-PAGE). Subsequently, proteins were transferred onto polyvinylidene difluoride (PVDF) membranes (Millipore, Bedford, MA, USA). After transfer, the membranes were blocked with 5% fat-free milk for 1 h at RT and incubated with either anti-cleaved Caspase-3 antibody (1:1000 dilution) (#G7481, Promega, Beijing, China), anti-Cytochrome C antibody (1:1000 dilution) (#ab76107, Abcam, Shanghai, China), anti- NFκB antibody (1:1000 dilution) (#8242S, Cell Signaling Technology, Shanghai, China), anti-TNFα antibody (1:1000 dilution) (#AF-510-NA, R&D Systems, Minneapolis, MN, USA), anti-eNOS antibody (1:1000 dilution) (#ab76198, Abcam), anti-CIC-3 antibody (1:1000 dilution) (#45,075 Signalway Antibody) with gentle agitation at 4°C overnight. Membranes were then washed for 15 min and incubated with horseradish peroxidase (HRP)-conjugated goat anti-rabbit secondary antibody (1:3000 dilution) (Kangchen Biotech, shanghai, China) for 90 min at RT. Then the membranes were washed three times for 15 min. The immunoreactive signals of target proteins were visualized by the ECL (enhanced chemiluminescence) detection. In order to identify the target protein expression, the X-ray films were scanned and analyzed by Image J 1.47i software [31,32]. This experiment was repeated three times.

### Identification of the concentration of chloride

N-(Ethoxycarbonylmethyl)-6-methoxyquinolinium bromide (MQAE) probe (Beyotime Biotechnology) was used to identify the concentration of chloride in bEnd3 cells. Briefly, 10mM MQAE was added into Krebs-HEPES buffer (20 mM HEPES, 128 mM NaCl, 2.5 mM KCl, 2.7 mM CaCl2, 1 mM MgCl2, 16 mM glucose, pH 7.4) to make up the work solution. Next, 1 × 10^8^/ml cells were incubated with the work solution for 1 h at RT in dark. After washing with Krebs-HEPES buffer five times, cells were analyzed by flow cytometry (FACSARIA, BD Biosciences, Franklin Lake, NJ, USA). This experiment was repeated three times.

### Flow cytometric analysis for programmed cell death

First, bEnd3 cells plated in 24-well plates (1 × 10^6^ cells/well) were treated by HG, Ang- [1–7] or siRNA. Then cells from each group were collected and washed twice with incubation buffer (10 mmol/L HEPES/NaOH, pH 7.4, 140 mmol/L NaCl, 5 mmol/L CaCl2). Next, cells were resuspended into 100 μl PBS containing 1.5 μg/ml Annexin V and moderate Propidium iodide (PI) (Thermo Scientific, Shanghai, China) and incubated at RT for 15 min in dark. After washing, cells were resuspended by incubation buffer and analyzed by flow cytometry (FACSARIA, BD Biosciences) [32,33]. This experiment was repeated three times.

### Analysis of mitochondrial potential

JC-1 dye (Invitrogen) was used to analyze the mitochondrial potential of bEnd3 cells. Briefly, bEnd3 cells were incubated with culture medium containing 1 µg/ml JC-1 dye for 15 min at 37℃. Next, cells were harvested and washed with PBS for 3 times. Subsequently, the stained cells were taking pictures using the BX50-FLA imaging system and analyzed by a flow cytometer (FACSARIA, BD Biosciences) [34]. This experiment was repeated three times.

### Examination of intracellular ROS generation

The oxidative conversion of cell-permeable oxidation of 2′, 7′-dichlorodihydrofluorescein diacetate (DCFH-DA) to fluorescent DCF was utilized to determine intracellular ROS generation in this study. First, bEnd3 cells were incubated with 10 µmol/L DCFH-DA solution in serum-free medium at 37°C for 30 min. Subsequently, cells were washed five times with PBS, and DCF fluorescence was measured by a flow cytometer (FACSARIA, BD Biosciences) [33]. This experiment was carried out three times.

### Detection of nitric oxide (NO)

The level of NO in bEnd3 cells were detected by Nitric Oxide (NO) assay Kit (Nitrate reductase method) (Nanjing Jiancheng Bioengineering Institute, Nanjing, Jiangsu, China) according to the manufacturer’s instructions. This experiment was carried out three times.

### Determination of intracellular glutathione (GSH)

First, 1 × 10^6^ bEnd3 cells were seeded in a 10 cm dish (Corning Inc., Corning, NY, USA). Next, cells were deproteinized using 5% 5-sulfosalicylic acid solution. Subsequently, the cellular level of GSH was identified by Glutathione Assay Kit (Sigma-Aldrich, St. Louis, MO, USA) according to the manufacturer’s protocol. This experiment was carried out three times

### ELISA for detection of interleukin (IL)-1β, IL-6 and IL-8 in the culture supernatant

First, 1 × 10^4^ bEnd3 cells were cultured in a well of the 96-well plate. After indicated treatments, the levels of IL-1β, IL-6 and IL-8 in the culture media were detected by ELISA kits according to the manufacturer’s instruction (Solarbio, Beijing, China) [33].

These experiments were repeated three times.

### Statistical analysis

All data in the present study were presented as the mean ± standard deviation (SD). The unpaired Student’s t-test was utilized for the comparation between two groups, while statistics among multiple groups were analyzed by One way ANOVA. *P* < 0.05 was considered to indicate a statistically significant difference.

## Results

This study aimed to identify whether Ang- [1–7] attenuated diabetic CVD through suppressing vascular endothelial injury by CIC-3. We hypothesized that Ang- [1–7] suppressed vascular endothelial cell apoptosis to alleviate HG-induced vascular endothelial injury through inhibiting CIC-3 via Mas receptor.

### Ang- [1–7] alleviates HG-induced injuries of bEnd3 cells

**Figure 1.**
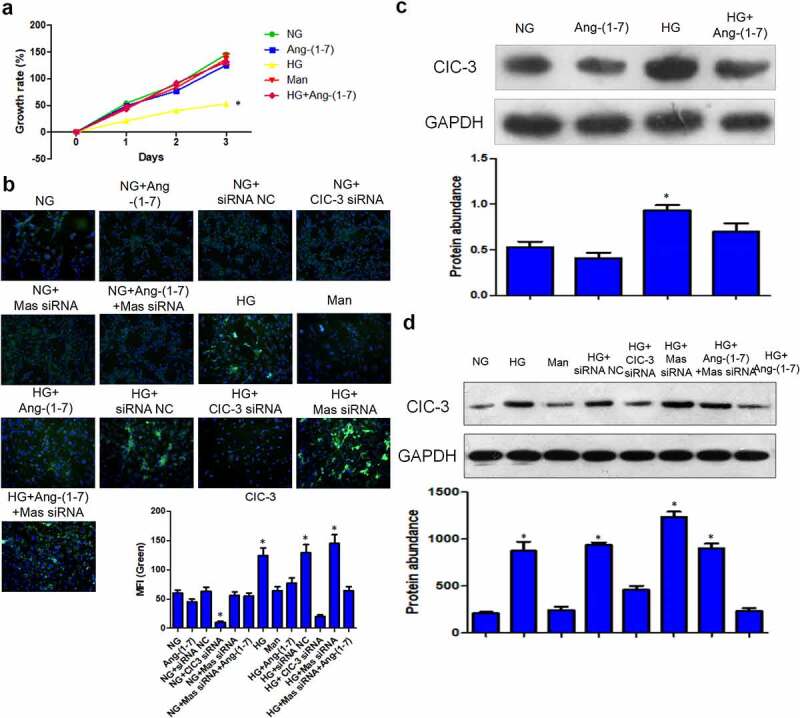
**Ang-** [1–7] **alleviates HG-induced injuries of bEnd3 cells through regulating CIC-3 expression via Mas receptor**. (a) Growth rate of bEnd3 cells in different groups. (b) Immunostaining analysis of CIC3 protein in bEnd3 cells. Green indicated CIC3 protein while blue indicated nucleus. (c) WB analysis of CIC3 protein level in bEnd3 cells treated with or without Ang- [1–7]. (d) WB analysis of CIC3 protein level in bEnd3 cells treated with or without siRNAs. * indicates P < 0.05 compared to NG group while # indicates P < 0.05 compared to HG group.

Results showed that high glucose suppressed bEnd3 cell proliferation while Ang- [1–7] protected bEnd3 cells against HG-induced growth suppression (Figure 1a). Moreover, Ang- [1–7] treatment under NG condition and mannitol treatment did not affect bEnd3 cell proliferation (Figure 1a). These data suggested that Ang- [1–7] ameliorated high glucose-induced injuries of bEnd3 cells.

### Ang- [1-7] reduces HG-induced CIC-3 expression through Mas receptor

To investigate downstream targets of Ang- [1–7], the expression of CIC-3 was detected by immunofluorescence and WB in bEnd3 cells. HG dramatically induced CIC-3 expression compared to that in NG-treated cells, while Ang- [1–7] reversed the effect of HG on the expression of CIC-3 (Figure 1b-d). Ang- [1–7] usually regulates downstream targets through activating Mas receptor [18,21]. Results revealed that silence of Mas abolished the effect of Ang- [1–7] on HG-induced CIC-3 expression (Figure 1b-d). In addition, the expression of CIC-3 was not regulated by mannitol and Ang- [1–7] and Mas in bEnd3 cells under NG condition (Figure 1 B). Moreover, CIC-3 siRNA could effectively reduce CIC-3 expression both in NG and HG-treated cells (Figure 1b-d). Therefore, Ang- [1–7] downregulated CIC-3 expression via Mas receptor in bEnd3 cells cultured with HG.

### Ang- [1-7] decreases the concentration of chloride in bEnd3 cells under HG condition

**Figure 2.**
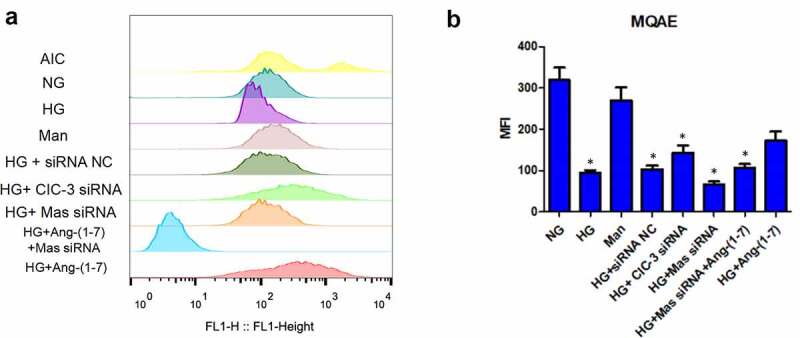
**Ang-** [1–7] **decreases the concentration of chloride in bEnd3 cells under HG condition**. (a) Chloride level in bEnd3 cells detected by MQAE probe using a flow cytometer. (b) Quantification of chloride level in bEnd3 cells detected by MQAE probe. * indicates P < 0.05 compared to NG group while # indicates P < 0.05 compared to HG group.

To further identify the effect of Ang- [1–7] on CIC-3, the concentration of chloride in bEnd3 cells was measured by MQAE. The fluorescence intensity of MQAE was decreased proportionally with the increase of chloride ion in cells. Compared to NG, HG significantly increased the concentration of chloride (Figure 2a-b). However, Ang- [1–7] treatment and CIC-3 silence alleviated the increase of chloride caused by HG (Figure 2a-b). Furthermore, silence of Mas neutralized the effect of Ang- [1–7] on the increase of chloride (Figure 2a-b). In addition, the concentration of chloride in bEnd3 cells was not modified by mannitol and siRNA NC (Figure 2a and b). Above data suggested that Ang- [1–7] suppressed the function of CIC-3 through Mas receptor under HG condition.

### Ang- [1-7] suppresses HG-induced bEnd3 cell apoptosis through inhibiting CIC-3 via Mas

**Figure 3.**
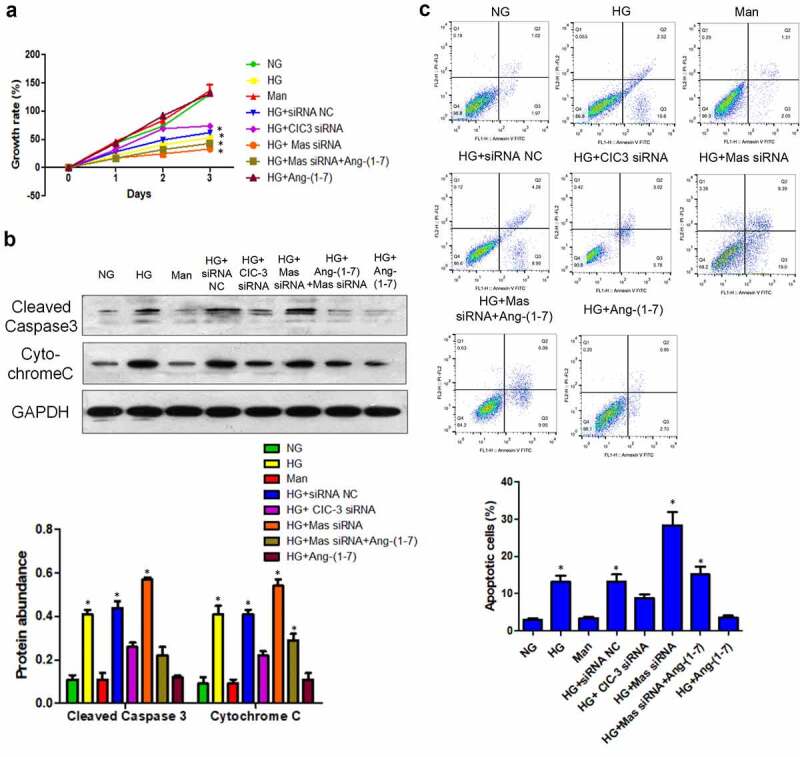
**Ang-** [1–7] **inhibits HG-induced bEnd3 cell apoptosis through suppressing CIC-3 via Mas**. (a) Growth rate of bEnd3 cells in different groups. (b) WB analysis of cleaved caspase3 and cytochrome C protein levels in bEnd3 cells. (c) Flow cytometric analysis of apoptosis in bEnd3 cells. * indicates P < 0.05 compared to NG group while # indicates P < 0.05 compared to HG group.

Consistent to the effect of Ang- [1–7] on bEnd3 cell proliferation, CIC-3 silence enhanced the growth of bEnd3 cell cultured with HG (Figure 3a). In addition, knockdown of Mas offset the impact of Ang- [1–7] on the HG-induced suppression of bEnd3 cell proliferation (Figure 3a). To identify whether Ang- [1–7] promoted bEnd3 cell proliferation through inhibiting apoptosis, Cleaved Caspase 3, CytochromeC and Annexin V were used for following experiments. Results showed that HG increased the level of cleaved Caspase 3, CytochromeC and the number of apoptotic cells, whereas Ang- [1–7] and CIC-3 silence ameliorated the HG-induced increasement of cleaved Caspase 3, CytochromeC and the number of apoptotic cells (Figure 3b-c). Moreover, silence of Mas abolished the effect of Ang- [1–7] on HG-increased cleaved Caspase 3, CytochromeC and apoptotic cells (Figure 3b-c). In addition, the apoptosis of bEnd3 cells was not regulated by mannitol and siRNA NC (Figure 3b-c). Thus, Ang- [1–7] reduced bEnd3 cell apoptosis caused by HG through suppressing CIC-3 via Mas receptor.

### Ang- [1-7] ameliorates HG-induced endothelial injury through downregulating CIC-3 via Mas in bEnd3 cells

**Figure 4.**
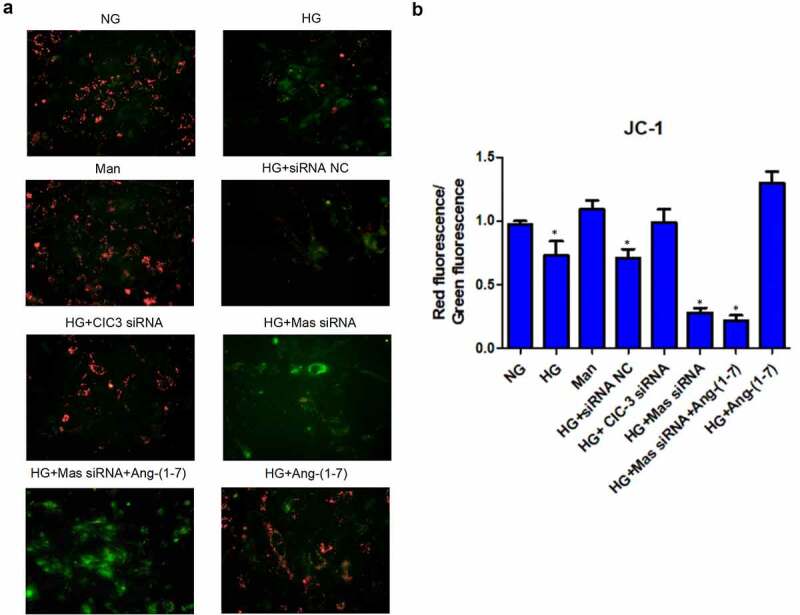
**Ang-** [1–7] **ameliorates HG-reduced mitochondrial potential through downregulating CIC-3 via Mas in bEnd3 cells**. (a) JC-1 dye-stained bEnd3 cells were analyzed for the mitochondrial potential. (b) Quantification of fluorescence for JC-1 dye detected by a flow cytometer. * indicates P < 0.05 compared to NG group while # indicates P < 0.05 compared to HG group.

**Figure 5.**
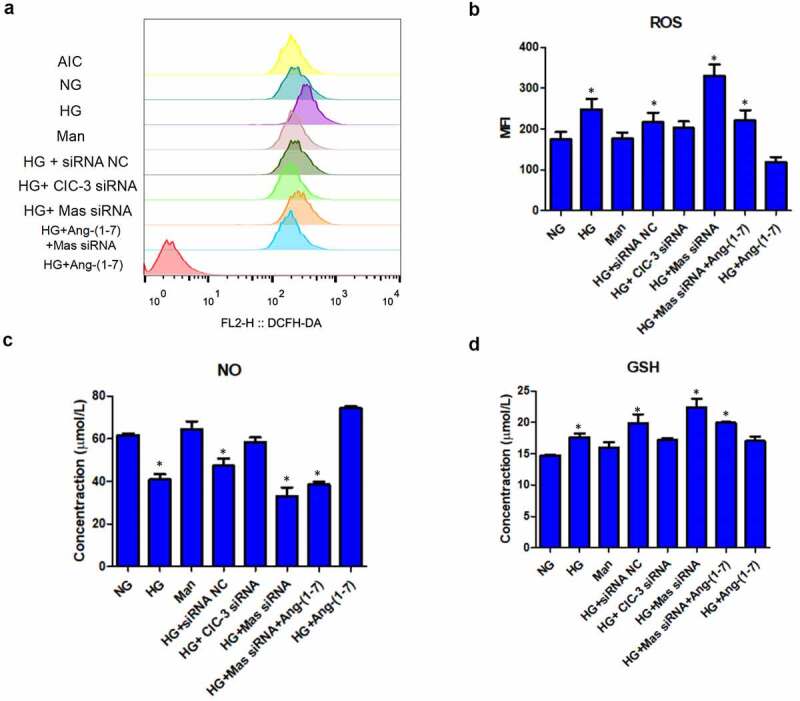
**Ang-** [1–7] **ameliorates HG-induced endothelial injury through downregulating CIC-3 via Mas in bEnd3 cells**. (a) ROS level in bEnd3 cells detected by DCFH-DA probe using a flow cytometer. (b) Quantification of ROS level in bEnd3 cells detected by DCFH-DA probe. (c) Quantification of NO level in bEnd3 cells. (d) Quantification of GSH level in bEnd3 cells. * indicates P < 0.05 compared to NG group while # indicates P < 0.05 compared to HG group.

Above data had revealed that Ang- [1–7] protected bEnd3 cells against HG. To further explore whether Ang- [1–7] protected bEnd3 cells against HG-induced endothelial injury, several markers of endothelial injury were detected. The mitochondrial membrane potential was indicated by JC-1 dye staining. Results showed that HG triggered mitochondrial depolarization revealed by the decrease of red/green fluorescence intensity ratio while Ang- [1–7] and CIC-3 silence preserved HG-induced mitochondrial depolarization (Figure 4a and b). What`s more, silence of Mas abolished the effect of Ang- [1–7] on HG-induced mitochondrial depolarization (Figure 4a and b). HG-induced mitochondrial depolarization subsequently resulted in the increase of ROS [35] (Figure 5a-b). Ang- [1–7] and CIC-3 silence alleviated HG-induced ROS whereas knockdown of Mas offset the effect of Ang- [1–7] on HG-increased ROS (Figure 5a-b).

**Figure 6.**
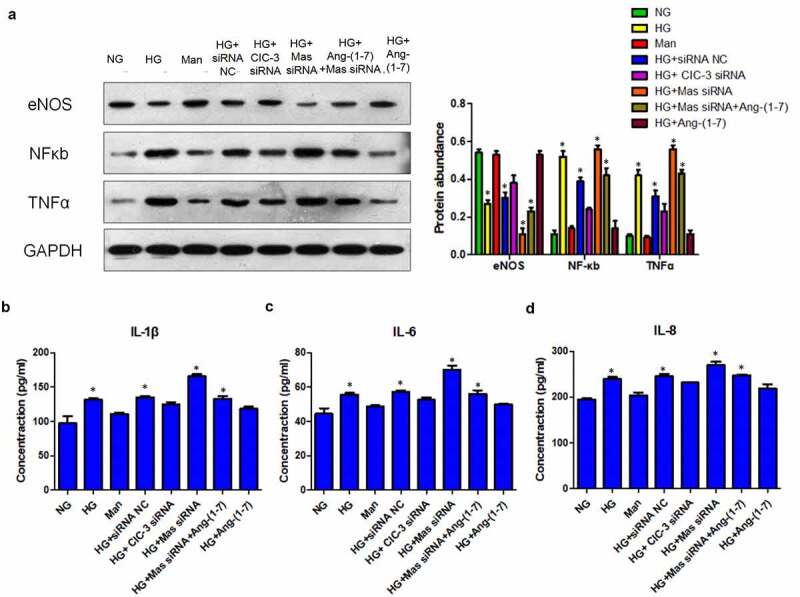
**Ang-** [1–7] **preserves HG-induced endothelial injury through suppressing cytokine production via Mas-CIC3 axis in bEnd3 cells**. (a) WB analysis of eNOS, NF-κb, TNFα protein levels in bEnd3 cells. (b) Levels of released IL-1β, IL-6 and IL-8 in bEnd3 cells. * indicates P < 0.05 compared to NG group while # indicates P < 0.05 compared to HG group.

Both NO and GSH play protective roles in endothelial injury [36,37]. HG dramatically reduced the level of NO and nitric oxide synthase eNOS in bEnd3 cells while Ang- [1–7] and CIC-3 silence preserved HG-reduced NO and eNOS (Figures 5C, 6A). However, silence of Mas neutralized the effect of Ang- [1–7] on the increase of NO (Figures 5C, 6A). In addition, Ang- [1–7] and CIC-3 did not modify the production of GSH (Figure 5d). Mannitol and siRNA NC did not modulate the mitochondrial membrane potential, ROS, NO, eNOS and GSH in bEnd3 cells (Figure 4a-B, 5A-D, 6A). These results together suggested that Ang- [1–7] ameliorates HG-induced endothelial injury through downregulating CIC-3 via Mas in bEnd3 cells.

### Ang- [1-7] preserves HG-induced endothelial injury through suppressing cytokine production via Mas-CIC3 axis in bEnd3 cells

Previous studies have indicated that cytokines can induce endothelial injury [38,39]. Results showed that HG upregulated the expression of NFκb, TNFα and enhanced productions of IL-1β, IL-6 and IL-8 (Figure 6a-d). Ang- [1–7] and CIC-3 silence reversed the effect of HG-induced NFκb, TNFα, IL-1β, IL-6 and IL-8 (Figure 6a-d). Moreover, silence of Mas offset the effect of Ang- [1–7] on the reduce of NFκb, TNFα, IL-1β, IL-6 and IL-8 under HG condition (Figure 6a-d). In addition, Mannitol and siRNA NC did not regulate the expression of NFκb, TNFα and enhanced productions of IL-1β, IL-6 and IL-8 (Figure 6a-d). Therefore, Ang- [1–7] may preserve HG-induced endothelial injury through suppressing cytokine production via Mas-CIC-3 axis in bEnd3 cells.

## Discussion

Diabetic CVD is the leading cause of deaths in patients with DM [5,6]. To date, there are limited effective medical therapies for diabetic CVD. Here, we found that Ang- [1–7] ameliorated high glucose-induced vascular endothelial injury through the inhibition of CIC-3 to reduce HG-induced ROS and cytokine. These results suggested that Ang- [1–7] may preserve diabetic CVD through suppressing HG-induced vascular endothelial injury.

Our results indicated that Ang- [1–7] attenuated HG-induced CIC-3 expression through Mas receptor for the first time. Numerous studies have revealed that Ang- [1–7] alleviates cardiovascular dysfunction including vascular endothelial injury through activating Mas receptor [18,21,40]. In addition, Ang- [1–7] drives the effect of anti-inflammatory via Mas receptor [41,42]. However, the mechanism how Ang- [1–7]/Mas axis downregulated CIC-3 was not clear.

A recent study has revealed that phosphorylation of ClC-3 by Rho-kinase 2 (ROCK2) is essential for Ang2-mediated vascular remodeling in hypertension [43]. In addition, phosphorylation/dephosphorylation plays a critical role in regulating ClC-3 channels [44]. By contrast, Ang- [1–7] attenuates Ang2-induced signaling through the dephosphorylation of Ang2-phosphorylated extracellular signal-related kinase (p-ERK1/2) via Mas receptor [45]. Numerous studies have indicated that dephosphorylation enhances protein degradation. For example, dephosphorylation by Small C-terminal Domain Phosphatase 1 (SCP1) accelerates the degradation of Twist1 protein [46]. Similarly, phosphates PH domain leucine-rich repeat protein phosphatase (PHLPP) induces the destabilization and subsequent degradation of protein kinase C (PKC) [47]. Moreover, inositol hexakisphosphate 6 (InsP6)-kinases enhance cytosolic InsP6 degradation through dephosphorylating InsP6 [48]. Therefore, Ang- [1–7] may reduce CIC-3 expression through the dephosphorylation-dependent protein degradation via Mas receptor.

HG-induced mitochondrial depolarization and subsequent ROS production promote vascular endothelial injury [49]. In this study, we found that Ang- [1-7] reduced mitochondrial depolarization and ROS under high glucose condition by suppressing CIC-3 via Mas receptor. Previous studies have demonstrated that CIC-3 promotes Ang2-induced mitochondrial depolarization and ROS production by NADPH oxidase to trigger the apoptosis of vascular endothelial cell [27,29,50]. All these studies suggested that Ang- [1–7] may attenuate HG-induced mitochondrial depolarization and subsequent ROS production through CIC-3/NADPH oxidase axis via Mas receptor.

By contrast to ROS, NO protects cells against endothelial injury and Ang- [1–7] usually exerts protective effects through NO [36,51,52]. This study revealed that HG decreased NO level by CIC-3 whereas Ang- [1–7] elevated NO production through suppressing CIC-3 via Mas. However, the mechanism under which CIC-3 inhibits NO production has not yet been elucidated. A previous study has found that activation of the ClC-3 leads to the inhibition of PI3K/Akt signaling pathway [53]. In contrast, the PI3K signaling-mediated NO production contributes to the protective effects of Ang- [1-7] on cardiovascular dysfunction [54]. Thus, CIC-3 may reduce NO production through suppressing PI3K/Akt signaling pathway.

Besides attenuating HG-induced ROS production, Ang- [1–7] also reduced HG-induced cytokine through the inhibition of CIC-3 via Mas receptor. It is well known that Ang- [1–7] protects against vascular endothelial injury through the effects of anti-inflammatory [18]. However, studies associated with the relationship between CIC-3 and cytokine are limited. A previous study has indicated that CIC-3 is required for cytokine activation in vascular smooth muscle cells [55]. In addition, a recent study has revealed that inhibition of CIC-3 abrogates lipopolysaccharide (LPS)-induced release of inflammatory cytokines through suppressing TLR4 pathway [56]. Conversely, Ang- [1–7] drives protective effects against LPS-induced lung injury through inhibiting TLR4 pathway [57]. These studies together suggested that Ang- [17] may exert anti-inflammatory effect through reducing cytokine production by blocking CIC-3/TLR4 pathway under HG condition.

## Conclusions

In summary, this study revealed that Ang- [1-7] suppressed vascular endothelial cell apoptosis to alleviate HG-induced vascular endothelial injury through inhibiting CIC-3 via Mas receptor, and Ang- [1-7] and CIC-3 may be novel targets for drugs against diabetic CVD.

## Highlights

- Ang-(1-7) ameliorates HG-induced vascular endothelial injury.
- Ang-(1-7) suppresses HG-induced vascular endothelial cell apoptosis through inhibiting CIC-3 via Mas receptor.
- Ang-(1-7) alleviates HG-induced vascular endothelial injury by the inhibition of CIC-3 via Mas receptor.
- Ang-(1-7) may protect diabetic CVD by suppressing HG-induced vascular endothelial injury.
